# Contrasting responses to Pleistocene climate changes: a case study of two sister species *Allium cyathophorum* and *A. spicata* (Amaryllidaceae) distributed in the eastern and western Qinghai–Tibet Plateau

**DOI:** 10.1002/ece3.1449

**Published:** 2015-03-10

**Authors:** Xinyu Wang, Yuanshuo Li, Qianlong Liang, Lei Zhang, Qian Wang, Huan Hu, Yongshuai Sun

**Affiliations:** MOE Key Laboratory for Bio-Resources and Eco-Environment, College of Life Science, Sichuan UniversityChengdu, 610065, China

**Keywords:** *A. spicata*, *Allium cyathophorum*, genetic diversity, Pleistocene climate changes, population size, Qinghai–Tibet Plateau, species divergence

## Abstract

It has been hypothesized that species occurring in the eastern and the western Qinghai–Tibet Plateau (QTP) responded differently to climate changes during the Pleistocene. Here, we test this hypothesis by phylogeographic analysis of two sister species, *Allium cyathophorum* and *A. spicata*. We sequenced two chloroplast DNA (cpDNA) fragments (*acc*D*-psa*I and the *rpl*16 intron) of 150 individuals, and the nuclear (ITS) region of 114 individuals, from 19 populations throughout the distributional ranges of these species. The divergence between the two species was dated at 779 - 714 thousand years before the present and was likely initiated by the most major glaciation in the QTP. Analysis of chlorotype diversity showed that *A. spicata*, the species occurring in the western QTP, contains much lower genetic diversity (0.25) than *A. cyathophorum* (0.93), which is distributed in the eastern QTP. Moreover, multiple independent tests suggested that the *A. spicata* population had expanded recently, while no such expansion was detected in *A. cyathophorum*, indicating a contrasting pattern of responses to Pleistocene climate changes. These findings highlight the importance of geographical topography in determining how species responded to the climate changes that took place in the QTP during the Pleistocene.

## Introduction

Responses to climate changes during the Pleistocene have previously been investigated for many plant and animal species in the Qinghai–Tibet Plateau (QTP) and other regions using phylogeographical analyses (Hewitt 2000; Arbogast and Kenagy 2001; Hickerson et al. 2010; Sandel et al. 2011; Liu et al. 2012). Some of these studies have revealed that the genetic diversity within a species was inevitably affected by climate changes in the QTP during the Pleistocene (Wang et al. 2009; Jia et al. 2011; Qiu et al. 2011). Many of the species distributed in the eastern QTP have been hypothesized to contain higher genetic diversity than species occurring the western QTP (Liu et al. 2012, 2014), indicating that geological topography may have played a role in determining the responses of different species to Pleistocene climate changes, such as the responses in genetic diversity and range size (Bellard et al. 2012). However, these comparative analyses have always focused on taxa with different life histories and/or biological preference. These biological factors have been hypothesized to be among the main drivers of genetic diversity in some taxa (Romiguier et al. 2014). Comparisons to date between closely related species which share a common life history and biological preference are rare, and thus, there is a need to conduct such studies in order fully to understand the effects of Pleistocene climate changes on species in the QTP. Here, we report the findings of a study that investigates the responses to these climate changes in a pair of sister species, *Allium cyathophorum* Bur. & Franch. and *A. spicata* Prain, which are distributed in the QTP and adjacent regions (Chen et al. 2000).

**Figure 1:**
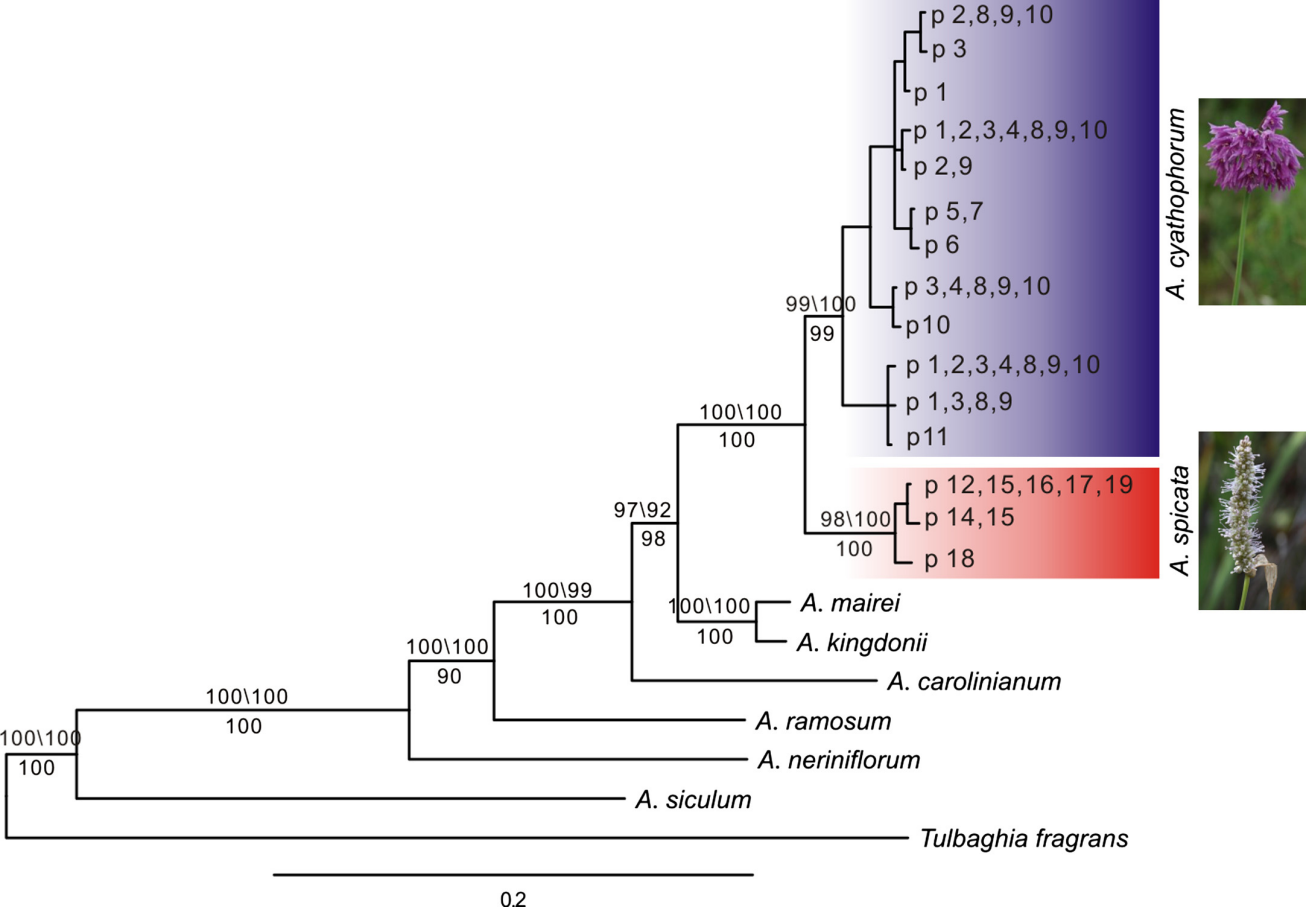
Phylogenetic tree reconstructed using the maximum-likelihood (ML) method on the basis of nrDNA ITS sequences. Bootstrap support values from MP\ML analyses are given above branches receiving >50% values in both analyses, while the corresponding posterior probabilities from Bayesian analyses are shown below branches. Photographs to the right show the inflorescences of *A. cyathophorum* and *A. spicata*.

*Allium cyathophorum* is distributed in the eastern QTP at an altitude of 2700–4600 m above sea level; *A. spicata* is distributed in the western QTP at a higher altitude, from 2900 to 4800 m above sea level. The area of overlap between them is limited, but the morphological characters of *A. spicata* are similar to those of *A*. *cyathophorum* with the exception of the type of inflorescence, as shown in Figure1 (Chen et al. 2000; Friesen et al. 2000). Several tens of sequence differences were found between the nuclear rDNA internal transcribed spacer (nrDNA ITS) region in *A. cyathophorum* and that in *A. spicata* (Friesen et al. 2000); assuming a rapid mutation rate for herbs, as discussed by (Kay et al. 2006), this finding indicates that the two species likely diverged before the last glacial maximum (LGM). However, this result may have been affected by incomplete lineage sorting due to the small sample size used for phylogenetic analysis (Kutschera et al. 2014; Rheindt et al. 2014). Developments in coalescent methods, such as approximate Bayesian computation (ABC), make it possible to take incomplete lineage sorting into account when dating the divergence between species (Csilléry et al. 2010). In addition, modeling the current and paleo-distributions of species can be used to examine responses to climatic changes during glacial periods (Guisan and Thuiller 2005; Elith and Leathwick 2009).

We sequenced two chloroplast DNA (cpDNA) fragments (*acc*D*-psa*I and the *rpl*16 intron) and the nuclear rDNA internal transcribed spacer (nrDNA ITS) region of 150 individuals collected from 19 populations throughout the ranges of *A*. *cyathophorum* and *A. spicata*. Using the population genetic sequence dataset and climatic variables for 59 locations, we aimed to (1) check whether *A*. *cyathophorum* and *A. spicata* are identified as sister species when employing multiple samples, and date the divergence between them, and (2) examine changes in the sizes of *A*. *cyathophorum* and *A. spicata* populations that occurred in response to Pleistocene climate changes. Finally, we discuss the contributions of geological topography and Pleistocene glacial cycles to changes in genetic diversity and population size.

## Material and Methods

### Population sampling

**Figure 2:**
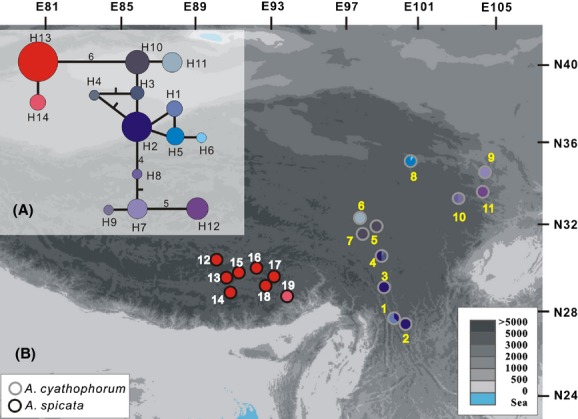
Network (A) and distribution (B) of chlorotypes within *A. cyathophorum* and *A. spicata*. (A) The numbers beside the lines indicate numbers of mutations, and the size of each circle is proportional to the frequency of the chlorotype. (B) The colors in each circle are proportional to the frequency of each chlorotype in each population. The colored circumferences of the circles indicate populations of *A. cyathophorum* (gray) and *A. spicata* (black).

We collected leaves of 150 individuals from 11 populations of *A. cyathophorum* and 8 populations of *A. spicata* (Table1, Fig.2). Within each population, the sampled individuals were separated by at least 100 m. Fresh leaves were dried in the field, and stored, in silica gel, and voucher specimens were deposited in the archives of Sichuan University (SCU). The altitude, latitude, and longitude of each collection center were measured using an Etrex GIS monitor (Garmin).

**Table 1:** Locations of 19 populations of *Allium cyathophorum* and *Allium spicata* and number (*N*) of chlorotypes per population

PC	Location	Alt (m)	Latitude	Longitude	Chlorotype (*N*)
*Allium cyathophorum*					
1	Zhonghoushan, YN	3360	N27°50′11″	E99°41′21″	H1(5), H2(3)
2	Wufengshan, YN	3470	N27°48′11″	E99°45′56″	H2(13)
3	Xiangcheng, SC	3400	N29°6′17″	E99°38′33″	H2(8)
4	Manigange, SC	3910	N31°55′35″	E99°11′59″	H2(4), H3(3), H4(1)
5	Xiaosumang, QH	3460	N32°11′10″	E97°14′54″	H10(10)
6	Xiaorongga, QH	3400	N32°16′56″	E96°16′4″	H11(6)
7	Maozhuang, QH	3520	N32°16′18″	E96°49′26″	H10(9)
8	Saizongsi, QH	3290	N35°33′13″	E99°50′42″	H5(6), H6(1)
9	Yangshalinchang, GS	2400	N34°49′39″	E103°40′23″	H7(8)
10	Langmusi, GS	3400	N34°5′33″	E102°37′53″	H8(1), H9(1)
11	Chuanzhusi, SC	3100	N34°47′3″	E103°37′54″	H12(7)
*Allium spicata*					
12	Dangxiong, XZ	4269	N30°26′23′’	E91°1′50′’	H13(5)
13	Qushui, XZ	3966	N29°14′13″	E90°37′52″	H13(17)
14	Langkazi, XZ	4460	N28°58′49″	E90°26′36″	H13(2)
15	Zhangnang, XZ	3567	N29°15′43″	E91°21′55″	H13(11)
16	Gongbujiangda, XZ	4249	N29°53′54″	E92°30′38″	H13(10)
17	Langxian, XZ	3203	N29°4′41″	E92°47′19″	H13(11)
18	Jiacha, XZ	3203	N29°4′37″	E92°47′18′	H13(2)
19	Langxian, XZ	3534	N28°54′01″	E93°23′51″	H14(6)

PC, population code; Alt, altitude; YN, Yunnan Province; SC, Sichuan Province; QH, Qinghai Province; GS, Gansu Province. XZ, Xizang Autonomous Region.

### DNA extraction, amplification, and sequencing

Total genomic DNA was extracted from the silica-dried leaves of each individual using the CTAB method (Doyle and Doyle 1990; Porebski et al. 1997). Two cpDNA fragments (the *acc*D*-psa*I intergenic spacer and the *rpl*16 intron) and the ITS region (including ITS1, 5.8S, and ITS2) were amplified and sequenced with primers used in previous studies (White et al. 1990; Hamilton 1999). Each polymerase chain reaction (PCR) was performed in a 25 *μ*L volume, containing 10–40 ng plant DNA, 50 mmol/L Tris-HCI, 1.5 mmol/L MgCl2, 250 *μ*g/mL BSA, 0.5 mmol/L dNTPs, 2 *μ*mol/L of each primer, and 0.75 unit of *Taq* polymerase. For the *accD-psaI* intergenic spacers, reactions were conducted with the following program: an initial denaturation at 94°C for 3 min, followed by 35 cycles of denaturation at 94°C for 1 min, annealing at 55°C for 50 sec, and extension at 72°C for 1 min plus a final extension at 72°C for 7 min, and all the products were held at 4°C until required for further processing (Small et al. 1998). For the *rpl*16 intron, reactions were conducted as follows: an initial denaturation at 94°C for 3 min, 37 cycles of denaturation at 94°C for 45 sec, annealing at 60°C for 55 sec, and extension at 72°C for 1 min 15 sec, with a final 7-min extension at 72°C (Small et al. 1998). For the ITS region, reaction conditions were as follows: an initial denaturation at 94°C for 5 min, followed by 36 cycles of denaturation at 94°C for 45 sec, annealing at 55°C for 55 sec, and extension at 72°C for 1 min 30 sec plus a final extension at 72°C for 7 min. PCR products were purified using a TIANquick Midi Purification Kit following the manufacturer's protocol (TIANGEN). Purified PCR products were directly sequenced using an ABI Prism BigDye Terminator version 3.1 Cycle Sequencing Kit and then separated on an ABI 3130 XL sequencer (Applied Biosystems, in Sichuan University, China.). Complete sequence alignments were obtained using the program Clustal X (Thompson et al. 1997; Larkin et al. 2007) and checked by eye in MEGA5 (Tamura et al. 2011). All sequences were deposited in GenBank (Accession Numbers: KP860949-KP860985).

### Phylogenetic analyses

To confirm the phylogenetic relationship of *A. cyathophorum* and *A. spicata* as sister species, we constructed a phylogenetic tree of eight species in the genus *Allium*, *A. siculum, A. neriniflorum, A. ramosum, A. carolinianum, A. kingdonii, A. mairei*, *A. cyathophorum,* and *A. spicata***,** using maximum-parsimony (MP), maximum-likelihood (ML), and Bayesian inference methods based on variations in the ITS sequences. *Tulbaghia fragrans* was used as outgroup taxon; the sequences for this species were downloaded from GenBank (Table S1). MP analysis was conducted by PAUP 4.10b (Swofford 2003), employing a heuristic search; obtaining the starting tree with the stepwise, treebisection-reconnection (TBR) branch swapping, steepest descent, and MulTrees and Collapse options selected; and setting no upper limit for the number of trees held in memory. Maximum-likelihood analyses were performed by PHYML 3.0 with 1000 bootstraps under the GTRIG model selected by jModeltest 2.0 (Guindon and Gascuel 2003; Darriba et al. 2012). MrBayes 3.1.2 was used for Bayesian inference analysis to find the optimal tree topography (Ronquist and Huelsenbeck 2003). Four runs were made, each to ten million generations, saving every 500th tree. Markov chain Monte Carlo convergence was explored by examining the potential scale reduction factor convergence diagnostics for all model parameters (Gelman and Rubin 1992). The posterior probabilities indicating support values for each branch were also estimated with a 25% “burn-in.”

### Population and phylogeographic analyses

We used DnaSP version 5.0 (Librado and Rozas 2009) to identify different chloroplast haplotypes. Indels were coded as single binary characters using Gapcoder (Young and Healy 2003). For each species, we calculated average gene diversity within populations (*H*_S_), total gene diversity (*H*_T_), and estimates of the population differentiation *G*_ST_ (Nei 1973) and *N*_ST_ based on the chloroplast dataset using the program PERMUT with 1000 permutations (Pons and Petit 1996). Genetic differentiation within and among populations within each species and differentiation between species were computed by analysis of molecular variance (AMOVA) using the program Arlequin version 3.0 (Excoffier et al. 2005). Significance was tested using 1000 random permutations.

To reconstruct the genealogy of cpDNA haplotypes from *A. cyathophorum* and *A. spicata*, we performed network analysis using TCS version 1.21 (Clement et al. 2000). The significance of isolation by distance between populations was assessed using Mantel tests with 1000 random permutations applied to matrices of pairwise population *F*_ST_ values and the natural logarithms of geographical distances for the two species together and separately (Rousset 1997). Pairwise *F*_ST_ values between populations were estimated using Arlequin version 3.0 (Excoffier et al. 2005); geographical distances between populations were calculated using an online tool (www.indo.com/distance/).

To examine whether the uplifts of the QTP had promoted divergence between *A. spicata* and *A. cyathophorum*, we estimated their divergence time using BEAST 1.8 (Drummond et al. 2012) on a basis of cpDNA sequences. The UPGMA tree is set as the starting tree. The mutation rate is assumed to be 1.5 × 10^−9^ s/s/y (Yamane et al. 2006). To get a robust estimation of divergence time, we used an interval from 1 × 10^−9^ s/s/y to 1 × 10^−8^ s/s/y and this prior is assumed to follow the uniform distribution implemented in BEAST. A total of 10,000,000 generations are set in Markov chain, and the genealogies are sampled per 1000 generation. Other parameters were set as default. Finally, the first 1,000,000 generations were used as burin-in and the remaining were used to summarize the divergence time with Tracer. The ESS values of all parameters are larger than 300.

### Tests of population expansion

In order to investigate whether recent population expansion events had taken place in *A. cyathophorum* and *A. spicata*, mismatch distribution analysis based on pairwise nucleotide differences within samples (Rogers and Harpending 1992) was implemented using Arlequin version 3.0 (Excoffier et al. 2005). Both the demographic expansion model and the spatial expansion model were used. Multimodal or random and irregular distributions of pairwise differences can be the characteristic of populations that have been stable for a long time, whereas populations that have experienced sudden demographic expansion should theoretically display smooth unimodal distributions (Slatkin and Hudson 1991; Rogers and Harpending 1992). DnaSP was used to provide graphic representations. The sum of squared deviations (SSD) and Harpending's raggedness index were used to test the validity of the demographic expansion model.

Tajima's *D* value and Fu's *F*_S_ value were also calculated, and significance was determined from 1000 resamplings using Arlequin version 3.0 (Excoffier et al. 2005). We used LAMARC 2.1.3 (Kuhner 2006) to assess the goodness of fit of the exponential growth model characterized by the equation (*θ*_t_ = *θ*_initial_ – *g*^t^) to our chloroplast DNA variations and to compute maximum-likelihood-based estimators of the exponential growth rate (*g*). The Markov chain Monte Carlo approach, which takes into account genealogical relationships between sequences, was used. We obtained the final estimates by running the program with 10 short chains of 10,000 steps and two long chains of 200,000 steps (sampling increment 20). The 95% confidence intervals for *g* were assessed by LAMARC and were used to test the significance of results. Three independent runs were performed to ensure the robustness of results.

### Test of speciation models

**Figure 3:**
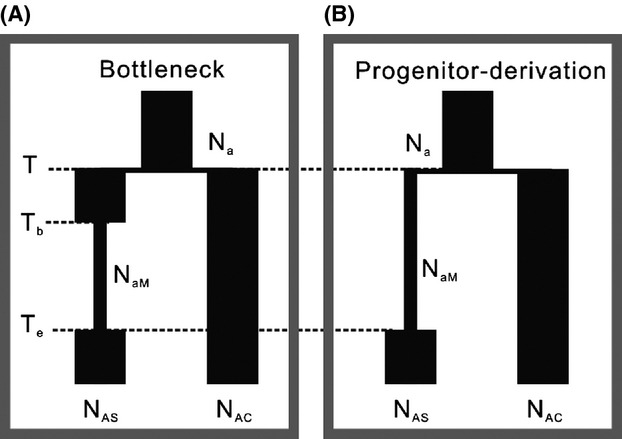
Models for the origin of *A. cyathophorum* and *A. spicata*. For Models A and B, *T* represents time of divergence between *A. cyathophorum* and *A. spicata* in generations; *N*_AC_ and *N*_AS_ represent current effective population sizes of *A. cyathophorum* and *A. spicata,* respectively; *N*_a_ represents the effective population size of their common ancestor; and *N*_aM_ represents the ancestral population size of *A. spicata*. For model A, *T*_b_ and *T*_e_ represent the beginning and end times of the hypothesized period of bottleneck for *A. spicata*; for model B, *T*_e_ represents instantaneous time of expansion of *A. spicata* from a population with small population size.

The population analyses described above revealed that genetic diversity within the alpine endemic species *A. spicata* is much lower than that within *A. cyathophorum*. This result, together with the phylogenetic tree, indicated that *A. spicata* may have originated from a founder population of *A. cyathophorum*. We tested two hypotheses relating to this pattern of intraspecific diversity observed in the two sister species (Fig.3) based on the cpDNA variations: (1) *A. spicata* and *A. cyathophorum* diverged before the LGM and experienced bottlenecks during the glacial period, and (2) *A. spicata* is derived from a local dispersal of an *A. cyathophorum* population after the LGM and has established its current distribution through recent expansion. The model comparison was performed using approximate Bayesian computation approach implemented in ABCtoolbox by simulating 350,000 datasets per model (Wegmann and Excoffier 2010; Wegmann et al. 2010). The model with the better fit to the observed statistics has a high probability of generating the observed pattern of molecular variations and thus can be used to compare different evolutionary scenarios (Beaumont et al. 2002). In this study, we used 15 statistics to summarize the pattern of molecular variation, including the number of haplotypes within each species, the number of polymorphic sites within each species, the number of private polymorphic sites for each species, the number of pairwise differences, Tajima's *D* (Tajima 1989), Fu's *F*_S_ (Fu 1997), pairwise *F*_ST_, the number of pairwise differences between species, and the total number of polymorphic sites. These statistics were computed by Arlequin version 3.0 (Excoffier et al. 2005) for both observed and simulated datasets. Simulated datasets were generated using a standard algorithm in ABCsampler, part of the ABCtoolbox software package, and fastsimcoal (Excoffier and Foll 2011). When simulating, the mutation rate is assumed to be 1.5 × 10^−9^ s/s/y (Yamane et al. 2006) and the prior of each parameter is assumed to follow the uniform distribution (Table S2). To decrease the redundancy of statistics, we first computed 11 partial least squares (PLS) components from the total of 15 summary statistics using an R script (Wegmann et al. 2010) and 10,000 datasets simulated by fastsimcoal. For each model, we retained the 3500 simulated datasets that were closest to the observed dataset after converting to PLS components and we applied the regression adjustment general linear model to compute marginal density and to generate posterior distributions of all parameters. The Bayesian factor was calculated using the marginal density of each model and used to determine which model fitted our dataset better.

### Modeling of species distribution

If *A. cyathophorum* and *A. spicata* diverged earlier than the LGM, these two species presumably survived in the QTP and adjacent regions during the LGM. We reconstructed species distributions to examine whether, and if so where, the likely LGM refugia of these two species existed in the QTP using paleo-climate data and MAXENT version 3.3.3k (Phillips et al. 2006; Phillips and Dudík 2008). Coordinates from 37 localities for *A. cyathophorum* and 22 for *A. spicata* were obtained from field observation and herbarium records (Table S3). Current environmental data and paleo-climate data (CCSM model) comprising 19 variables, and altitude data, were downloaded from WORLDCLIM (www.worldclim.org) with resolutions of 2.5′ (Hijmans et al. 2005). We examined pairwise correlations among the 20 variables within each species distribution and also across the two species. The seven environmental variables (altitude, mean diurnal range, minimum temperature of coldest month, temperature annual range, annual precipitation, precipitation of driest month, and precipitation seasonality) with pairwise Pearson's correlation coefficients of *r* ≤ 0.7 were used to reconstruct the species distributions, to minimize biased fitting of niche models. We first modeled the climate niches of two species separately using MAXENT with the default parameters and included 80% (20%) of the species records for training (testing) the model, and then, we projected the niches to the paleo-climate layers. Graphics were drawn using DIVA–GIS 7.5 (http://www.worldclim.org/).

## Results

### ITS DNA variation

The length of the ITS sequence was from 578 bps to 586 bps across the 114 individuals from 19 populations. In total, we identified 12 ITS sequences in *A. cyathophorum* and 3 in *A. spicata*, indicating a lower level of genetic diversity in *A. spicata*. Phylogenetic analyses using PAUP, PhyML, and MrBayes revealed that the populations from *A. cyathophorum* and *A. spicata* were grouped into a single clade with high bootstrap support and posterior probability values (Fig.1). Thus, our ITS data supported the conclusion that *A. cyathophorum* is sister to *A. spicata*.

### CpDNA variation

The total length of the two chloroplast DNA sequences was from 1444 bp to 1452 bp across the 150 individuals in this study. Nucleotide substitutions, consisting of four transitions, six transversions, and seven indels, occurred at 17 sites within these two cpDNA regions. A total of 14 haplotypes were identified based on these polymorphisms, including 12 haplotypes (H1–H12) found in *A. cyathophorum* and two haplotypes (H13, H14) that were present exclusively in *A. spicata*, again indicating lower genetic diversity in *A. spicata*. Genealogical analyses of these 14 chlorotypes recovered two major clades, one comprising H1 to H12 and the other including the remaining two chlorotypes (Fig.2).

### Population and phylogeographic analyses

The intrapopulation chlorotype diversity values were 0.82 and 0.11 for *A. cyathophorum* and *A. spicata,* respectively. The total genetic diversity within species (*H*_T_) was 0.93 and 0.25 for *A. cyathophorum* and *A. spicata,* respectively (Table2). *N*_ST_ was significantly higher than *G*_ST_ for *A. cyathophorum* (*N*_ST_ = 0.955, *G*_ST_ = 0.755, *P *<* *0.05) but not for *A. spicata* (*N*_ST_ = 1.0, *G*_ST_ = 1.0), indicating significant phylogeographic structure in *A. cyathophorum*.

**Table 2:** Estimates of average genetic diversity within populations (*H*_S_), total genetic diversity (*H*_T_), interpopulation differentiation (*G*_ST_), and the number of substitution types (*N*_ST_) for haplotypes

Group	*H*_S_	*H*_T_	*G*_ST_	*N*_ST_
*Allium cyathophorum*	0.23	0.93	0.76	0.955*
*Allium spicata*	0	0.25	1	1^ns^
Total	0.13	0.85	0.85	0.988*

*P <* *0.05; ^ns^*P* > 0.05.

Based on AMOVA of the cpDNA sequence dataset, the variation between populations within species accounts for a very high proportion of the total variation for both *A. cyathophorum* (98%) and *A. spicata* (100%). In addition, significant differentiation between species was indicated by the high *F*_CT_ value (0.838, *P *<* *0.01; Table3). Mantel tests applied to each species separately suggested nonsignificant correlation between geographical and genetic distance (*A. cyathophorum*: *r*^2^ = 0.197, *P *=* *0.083; *A. spicata*: *r*^2^ = 0.106, *P *=* *0.250). However, when we combined the data from the two species, we detected significant correlation between geographical and genetic distance (*r*^2^ = 0.423, *P *=* *0.01), indicating that geographic barriers may have contributed to interspecific differentiation.

**Table 3:** Analysis of molecular variance (AMOVA) for populations of *A. cyathophorum* and *A. spicata* based on chloroplast haplotypes

Source of variation	df	SS	VC	V%	*F*-statistic
All samples					
Among species	1	1511.918	20.086	83.88	*F*_CT_ = 0.838*
Among populations within species	17	495.278	3.788	15.82	*F*_SC_ = 0.981*
Within populations	131	9.375	0.071	0.3	*F*_ST_ = 0.997*
Total	149	2016.553	23.946		
*Allium cyathophorum*					
Among populations	10	463.317	5.973	97.954	*F*_ST_ = 0.979*
Within populations	75	9.357	0.124	2.046	
Total	85	472.674	6.098		
*Allium spicata*					
Among populations	7	5.438	0.102	100	*F*_ST_ = 1.000*
Within populations	56	0	0	0	
Total	63	5.438	0.102		

df, degrees of freedom; SS, sum of squares; VC, variance components; V%, percent variation; *F*_ST_, differentiation among populations; *F*_SC_, differentiation among populations within species; *F*_CT_, differentiation among species; *, *P *<* *0.01, 1000 permutations.

**Figure 4:**
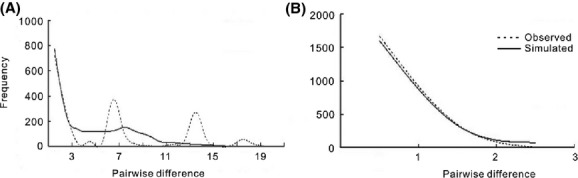
Mismatch distribution analysis for *A. cyathophorum* (A) and *A. spicata* (B) based on the spatial expansion model. The solid line shows simulated distributions for a putatively expanding population, and the dashed line represents the observed distribution of pairwise differences among samples.

Mismatch distribution analysis of sequence data from either *A. cyathophorum* or *A. spicata* suggested that the latter species has expanded recently, but the former species has not (Fig.4). The hypothesis of expansion in the case of *A. spicata* was also supported by a g test performed using LAMARC, which gave a positive growth rate value, and by the Harpending's raggedness index (*P *=* *0.43, Table4).

**Table 4:** Results of demographic analyses performed with multiple methods

Species	Mismatch distribution				Neutrality test		LAMARC
	*θ*_0_	*θ*_1_	SSD (*P*-value)	RAG (*P*-value)	Fu's *F*s (*P*-value)	Tajima's *D* (*P*-value)	Growth rate (95%CI)
*A. cyathophorum*	0.08	12.38	0.051 (0.060)	0.058 (0.020)	9.290 (1.000)	2.098 (0.970)	−137.972 (−680.856–258.900)
*A. spicata*	0.45	0.45	0.021 (0.130)	0.458 (0.430)	0.192 (0.286)	−0.232 (0.252)	10,393.18 (−5000.00–25,000.00)

*θ*_0_ and *θ*_1_, pre- and postexpansion populations sizes; SSD, sum of squared deviations; RAG, Harpending's raggedness index; Fu's *F*s and Tajima's *D* are shown with *P* values as above; CI, confidence interval.

### Test of speciation models

To test whether *A. spicata* originated from founder populations of *A. cyathophorum*, we constructed two speciation models (Fig.4) and compared them using the ABC approach. The results showed that the bottleneck model (model A in Fig.3) fits our data better than the hypothesized model B, which is of the progenitor derivation type (Bayes factor = 2.33), indicating that our data do not support founder speciation as an explanation for the origin of *A. spicata*. The low Bayes factor may be due to the limited extent of variation in our data.

Under the bottleneck model, the divergence time between the species was estimated at 779 (95% HPDI: 960 – 54) thousand years ago (Ka), and *A. spicata* underwent an extended period of bottleneck from 36 to 10 thousand years ago (Table5). Consistent roughly with this estimate by ABCtoolbox, the divergence between these two species was dated at around 714 Ka (95% HPDI: 1908 – 143 Ka) using BEAST 1.8. After the bottleneck period, the population size of *A. spicata* increased to 9821 (95% HPDI: 3162 – 51591) individuals from a smaller population with a size of 334 (95% HPDI: 32 - 7986) individuals. In contrast, the current population size of *A. cyathophorum* is 3.56 × 10^5^ (95% HPDI: 1.66 × 10^5^ – 6.08 × 10^5^) individuals, much greater than the *A. spicata* population size. In addition, under the progenitor derivation model, the divergence time was estimated at 808 (95% HPDI: 990 – 82) Ka, a date generally consistent with the estimate obtained under the bottleneck model.

**Table 5:** Posterior mode estimate and 95% highest posterior density interval (HPDI) for demographic parameters based on the nuclear multilocus sequence data. *T*, divergence time between *A. cyathophorum* and *A. spicata* in generations; *N*_AS_ and *N*_AC_, current population size of *A. cyathophorum* and *A. spicata*, respectively; *N*_a_*,* effective population size of the common ancestor; *N*_aM_, the ancestral population size of *A. spicata* during the bottleneck period from *T*_b_ (beginning time) to *T*_e_ (end time)

Parameter	*N*_a_	*N*_AC_	*N*_aM_	*N*_AS_	*T* (years)	*T*_b_ (years)	*T*_e_ (years)
Mode	133,870	355,630	334	9821	778,572	36,066	10,049
95% HPDI lower	6310	166,343	32	3162	53,932	10,886	1000
95% HPDI upper	978,952	608,012	7986	51,591	959,732	40,000	32,083

### Modeling of species distribution

**Figure 5:**
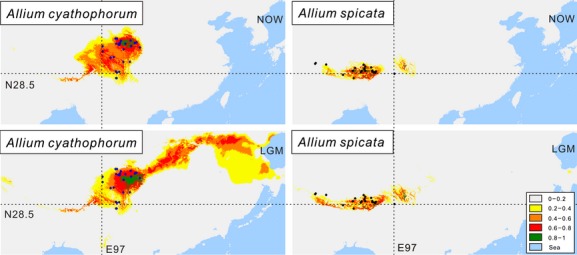
Climatic niche models for the present-day (top) and paleo-distribution models during the last glacial maximum (LGM) (bottom) for *A. cyathophorum* (left) and *A. spicata* (right) in the Qinghai–Tibet Plateau. The blue and black circles represent the sampling sites used in ecological niche modeling analyses for *A. cyathophorum* and *A. spicata*, respectively. The dotted lines depict longitude and latitude to facilitate a comparison between the geographical distributions of the two species at the two different time points. Colors represent bioclimatic suitability, from most suitable (red) to unsuitable (gray).

Areas under the ROC curve (AUC) had values of 0.991 and 0.995 for *A. cyathophorum* and *A. spicata,* respectively, indicating that differences varied greatly from random expectation when modeling the current species distributions. These modeled distributions indicate that *A. cyathophorum* has a wider distribution than *A. spicata*, a result consistent with our field observations (Fig.5). We further constructed the paleo-distributions of both species based on the climate dataset from WORLDCLIM and calculated the size of the range of overlap. Using a suitability probability of 0.75 as the threshold, the overlap in area between the paleo-distributions of *A. cyathophorum* and *A. spicata* is zero. In addition, we observed that the paleo-distribution seems to be wider than the current distribution for either species, indicating that the demographic histories of these species were complex and may have involved repeated range shifts during and/or after the LGM.

## Discussion

In this study, using a population genetic sequence dataset, we confirmed that *A*. *cyathophorum* is sister to *A*. *spicata*, a finding consistent with previous phylogenetic studies (Huang et al. 2014). Dating analyses using the ABC method suggested that the divergence between *A*. *cyathophorum* and *A*. *spicata* occurred 779–714 Ka, at the time of the most major glacial period (∽600–1200 Ka) in the QTP (Shi et al. 1998). Genetic diversity analysis of the two species showed that *A. spicata*, the species living in the eastern QTP, has lower diversity than its sister species, *A. cyathophorum*, which lives in the western QTP. Phylogeographic analysis suggested that the quantity and/or location of glacial refugia and the demographic histories of the two species contributed to this distinct pattern of intraspecific diversity. ABC analysis indicated that the population size of *A. spicata* during the LGM (334 individuals) was much smaller than its current population size (9821 individuals), in accordance with the results of mismatch distribution analysis and the *g* test which showed that recent population expansion had occurred in *A. spicata,* but not in *A. cyathophorum*. The present study highlights that species distributed in the western QTP responded to the Pleistocene climate changes in a different way from species distributed in the eastern QTP.

### Divergence between *A. cyathophorum* and *A. spicata*

The results of ABC analysis dated the divergence between *A*. *cyathophorum* and *A. spicata* at around 779 Ka (960–54 Ka; Table5) or 714 Ka estimated by ABCtoolbox and BEAST, respectively, when the most extensive glacial period, 1200-600 Ka, in the QTP was still in progress (Shi et al. 1998). This estimate suggests that the divergence was not triggered by the most recent extreme uplift of the QTP during the Pliocene and early Pleistocene, but probably resulted from a scenario as follow. During the middle Pleistocene prior to the divergence between *A*. *cyathophorum* and *A. spicata*, their common ancestor may have been distributed widely in the QTP, occupying the platform and the eastern edge. When the ice sheets advanced during the most extensive glacial period, these ancestral populations were restricted to multiple isolated refugia where they initiated the formation of novel species. The distinct environmental conditions of the western QTP probably spurred more extensive genetic differentiation and promoted the establishment of specialized niche and morphological characters in *A. spicata*, facilitated by genetic drift due to small population size in this species (Mayr 1942, 1970). The results of ABC analysis showed that the *A. spicata* population size during the bottleneck was less than 5% of the population size of *A*. *cyathophorum* (Table5). This indicates that after their initial divergence during the most extensive glacial period, the range and population size of *A. spicata* were reduced. In contrast, the *A*. *cyathophorum* population size did not greatly alter, indicating that this pair of sister species responded differently to climate changes after their divergence during the Pleistocene.

The glacial cycles during the late Pleistocene may have not only shifted species ranges but also triggered species divergence (Carstens and Knowles 2007). The reconstructed distribution of the two species during the LGM showed that the ranges of *A*. *cyathophorum* and *A. spicata* rarely overlapped, indicating the contribution that geological barriers made to further divergence (Hewitt 1996). Our results therefore suggest that the advance of the glaciers during the LGM further spurred the process of divergence by restricting these two species to isolated refugia.

### Extremely low genetic diversity in *A. spicata*

Based on the values for genetic diversity obtained in the present study and from the literature, *A. spicata*, which is endemic to the western QTP, has a lower level of diversity than the species that occur on the eastern edge of this Plateau. Haplotype diversity calculated on the basis of cpDNA variation in *A. spicata* (*H*_T_ = 0.25; Table2) is much lower than that in *A*. *cyathophorum* (*H*_T_ = 0.93), *A. przewalskianum* (*H*_T_ = 0.87) (Wu et al. 2010), and *A. wallichii* (*H*_T_ = 0.96) (Huang et al. 2014). The latter two species, *A. przewalskianum* and *A. wallichii*, are distributed in the eastern QTP and also in adjacent regions. The following explanations may account for this contrast in the pattern of genetic diversity.

Firstly, the scope/number of refugia available for *A. spicata* during the LGM may have had an effect. The geographic distribution of haplotypes showed that the common haplotypes H2 and H10 occurred exclusively in six populations of *A*. *cyathophorum* and each of the other ten chlorotypes occurred in only one population (Fig.2). The four chlorotypes H7, H8, H9, and H12, which bear three or more mutations when compared with the common chlorotypes H2 and H10, co-occurred in the three eastern populations (Table1). This distribution of chlorotypes indicated that at least three refugia for *A. cyathophorum* existed during the ice age. In contrast, in *A. spicata*, most populations were fixed for one haplotype, H7, and only one population was fixed for haplotype H8, indicating that the number of refugia for *A. spicata* was lower than that for *A. cyathophorum*. Reconstruction of paleo-distributions also showed that *A*. *cyathophorum* may have survived both in the eastern QTP and also in the east of China outside the QTP, during the LGM, spanning spatial scales greater than those of *A. spicata*.

Secondly, *A. spicata* experienced a long period of evolution with a small population size, which should have contributed to its low present-day genetic diversity. The signals of recent population expansion in *A. spicata* were detected on the basis of chloroplast DNA variations using several approaches including mismatch distributions and *g* test (Fig.4; Table4). The geographic distribution of haplotypes showed that a single haplotype (H13) is fixed in plants from seven populations of *A. spicata*, indicating that regional population expansion took place. Furthermore, a bottleneck event during the evolution of *A. spicata* was supported by model comparison analysis using the ABC approach. The estimated population size of *A. spicata* (334 individuals; Table5) during the bottleneck period (36–10 Ka) is much smaller than the current population size (9821 individuals). Historical demography, most likely caused by glacial cycles, therefore probably contributed to the lower level of genetic diversity in *A. spicata*.

In principle, the finding that *A. spicata* contained much less diversity than *A*. *cyathophorum* could be explained by whether *A. spicata* originated recently from a founder population of *A*. *cyathophorum*, as in the scenarios proposed for *Picea morrisonicola* (Zou et al. 2013). But this hypothesis is rejected by the results of ABC analysis, which gave a low Bayes factor value. If there had been recent founder speciation, the two species should share many alleles because of incomplete lineage sorting (Coyne and Orr 2004; Gavrilets 2004). However, fixed differences were found in both cpDNA and ITS sequence datasets. Thus, our data excluded the explanation that *A. spicata* originated recently from founder populations. However, the availability of multiple independent nuclear genomic loci would potentially offer higher power and accuracy with which to confirm the origin of *A. spicata*.

In summary, we investigated the genetic diversity and demographic histories of a pair of sister species, *A. spicata* and *A. cyathophorum*, which diverged from each other 779–714 Ka and are distributed in the western and eastern QTP, respectively. These two species experienced the same Pleistocene glaciations, but showed contrasting responses to these climate changes. Thus, our study has highlighted the fact that the differences in geographic topography between the western and eastern QTP played a major role in determining the contrast between the ways in which the two species responded.

## Supporting Information

**Table S1.** GI numbers of ITS sequences for species used as outgroups, obtained from GenBank. **Table S2.** Prior distributions about model parameters used in model comparisons. **Table S3.** The presence-only data (longitude and latitude) used in the ecological niche modelling.
