# Supplementary material for: Contrasting responses to Pleistocene climate changes: a case study of two sister species Allium cyathophorum and A. spicata (Amaryllidaceae) distributed in the eastern and western Qinghai–Tibet Plateau
Source: Ecol Evol. 2015 Mar 10;5(7):1513–24. doi: 10.1002/ece3.1449 (PMC4395180; doi:10.1002/ece3.1449)
Supplement: Supplementary file 1 — Table S1. GI numbers of ITS sequences for species used as outgroups, obtained from GenBank. Table S2. Prior distributions about model parameters used in model comparisons. Table S3. The presence-only data (longitude and latitude) used in the ecological niche modelling. [file ece30005-1513-sd1.doc]

# Supporting information

Table S1. GI numbers of ITS sequences for species used as outgroups, obtained from GenBank.

| Species | GI |
| --- | --- |
| *Allium kingdonii* | 11595362 |
| *Allium mairei* | 379323323 |
| *Allium siculum* | 11595453 |
| *Allium carolinianum* | 301130868 |
| *Allium neriniflorum* | 259186363 |
| *Allium ramosum* | 498424881 |
| *Tulbaghia fragrans* | 11595755 |

Table S2. Prior distributions about model parameters used in model comparisons.

| Parameters ^a^ | Prior ^b^ |
| --- | --- |
| LOG_N_AC | [4.8, 5.8] |
| LOG_N_AS | [3.5, 4.8] |
| LOG_NaM | [1.5, 4] |
| LOG_Na | [3.8, 6.2] |
| T | [500, 500000] |
| Tb | [500, 20000] |
| Te | [500, 20000] |

^a^ LOG refers here to the logarithm to base 10. AC and AS represents *A. cyathophorum* and *A. spicata*, respectively. N_a_ represents effective population sizes of the common ancestor of *A. cyathophorum* and *A. spicata*; N_aM_, the ancestral population size of *A. spicata*. ^b^ All priors are uniformly distributed.

## Table S3. The presence-only data (longitude and latitude) used in the ecological niche modelling.

| Site | Longitude (° E) | Latitude (° N) |  | Site | Longitude (° E) | Latitude (° N) |
| --- | --- | --- | --- | --- | --- | --- |
| AS1 | 97.069124 | 28.681698 |  | AC8 | 99.7060132 | 27.8230796 |
| AS2 | 86.999961 | 28.477712 |  | AC9 | 99.7992897 | 28.9320733 |
| AS3 | 91.162226 | 29.672376 |  | AC10 | 99.7992897 | 28.9320733 |
| AS4 | 81.621668 | 30.77789 |  | AC11 | 99.5635879 | 35.5586594 |
| AS5 | 89.081382 | 27.815088 |  | AC12 | 96.9991386 | 34.0554505 |
| AS6 | 93.440878 | 29.101786 |  | AC13 | 101.515989 | 34.5667966 |
| AS7 | 89.380043 | 29.814826 |  | AC14 | 97.0129681 | 33.0183078 |
| AS8 | 83.662595 | 30.484477 |  | AC15 | 97.0129681 | 33.0183078 |
| AS9 | 92.719872 | 29.293908 |  | AC16 | 101.134804 | 33.4254511 |
| AS10 | 91.347043 | 30.116478 |  | AC17 | 99.6734834 | 34.5688551 |
| AS11 | 91.348412 | 30.117236 |  | AC18 | 99.6734834 | 34.5688551 |
| AS12 | 93.106766 | 28.96475 |  | AC19 | 99.6734834 | 34.5688551 |
| AS13 | 88.8883853 | 29.2717159 |  | AC20 | 100.295423 | 34.4774993 |
| AS14 | 81.530583 | 30.63712 |  | AC21 | 100.295423 | 34.4774993 |
| AS15 | 90.443333 | 28.968889 |  | AC22 | 99.6734834 | 34.5688551 |
| AS16 | 90.618889 | 29.236944 |  | AC23 | 100.618093 | 35.1492929 |
| AS17 | 91.030556 | 30.43975 |  | AC24 | 99.6734834 | 34.5688551 |
| AS18 | 92.788333 | 29.077222 |  | AC25 | 100.4243 | 34.71472222 |
| AS19 | 92.788333 | 29.076944 |  | AC26 | 100.1439 | 34.4228 |
| AS20 | 91.365278 | 29.251944 |  | AC27 | 100.3638 | 34.3856 |
| AS21 | 93.385556 | 28.900278 |  | AC28 | 104.371458 | 33.7848009 |
| AS22 | 92.501667 | 29.898472 |  | AC29 | 102.521731 | 35.2025595 |
|  |  |  |  | AC30 | 103.35313 | 34.6926592 |
| AC1 | 101.1252344 | 30.9795408 |  | AC31 | 99.68921185 | 27.83651476 |
| AC2 | 98.5809124 | 31.8061203 |  | AC32 | 99.76556438 | 27.80311486 |
| AC3 | 100.2984059 | 29.0370076 |  | AC33 | 99.64270295 | 29.10462021 |
| AC4 | 99.7992897 | 28.9320733 |  | AC34 | 99.1997983 | 31.92655789 |
| AC5 | 102.364372 | 30.9990301 |  | AC35 | 99.835278 | 35.550278 |
| AC6 | 98.5809124 | 31.8061203 |  | AC36 | 103.6732625 | 34.82750612 |
| AC7 | 101.1252344 | 30.9795408 |  | AC37 | 102.6314857 | 34.09253384 |

AS, *A. spicata*; AC, *A. cyathophorum*.
